# Effect of Intravitreal Bevacizumab Injection before Panretinal Photocoagulation on the Prevention of Macular Edema Aggravation in Proliferative Diabetic Retinopathy

**DOI:** 10.3390/jcm9113772

**Published:** 2020-11-23

**Authors:** Wungrak Choi, Hyun Goo Kang, Eun Young Choi, Sung Soo Kim, Hyoung Jun Koh, Min Kim

**Affiliations:** 1 Department of Ophthalmology, Institute of Vision Research, Yonsei University College of Medicine, Seoul 03722, Korea; wungrakchoi@yuhs.ac (W.C.); HGKANG08@yuhs.ac (H.G.K.); eychoi@yuhs.ac (E.Y.C.); semekim@yuhs.ac (S.S.K.); hjkoh@yuhs.ac (H.J.K.); 2 Department of Ophthalmology, Gangnam Severance Hospital, 211 Eonju-ro, Gangnam-gu, Seoul 06273, Korea

**Keywords:** proliferative diabetic retinopathy, central macular thickness, panretinal photocoagulation, intravitreal bevacizumab, macular edema

## Abstract

Objective: To investigate the effects of intravitreal bevacizumab (IVB) injection before PRP on the prevention of macular edema (ME) in patients with PDR. Methods: This retrospective observational study included patients diagnosed with PDR treated by PRP with (combination group) or without (PRP alone group) preoperative IVB injection (1.25 mg/0.05 mL). The primary outcome measure was the change in the central macular thickness (CMT), while the secondary outcome measure was the change in visual acuity. Measurements were made before and at one, two, and three months after treatment. Results: In the PRP alone group, the mean baseline CMT of 277.8 μm increased to 290.4 μm at one month (*p* = 0.201) and 308.8 μm at two months (*p* = 0.002), followed by a decrease to 271.2 μm at three months (*p* = 0.437). In the combination group, the values at baseline and one, two, and three months after PRP were 322.9 μm, 295.4 μm (*p* = 0.002), 330.1 μm (*p* = 0.906), and 274.5 μm (*p* = 0.030), respectively. Visual acuity changes were comparable between the two groups at all time points. Conclusion: IVB injection before PRP leads to decreased CMT in comparison to CMT in patients with PRP alone.

## 1. Introduction

Diabetic retinopathy (DR) is one of the leading causes of vision loss in developed countries [1,2]. Studies have shown that macular edema (ME) and retinal neovascularization (NV) are among the most significant factors mediating vision loss in patients with DR [1,2,3]. The Diabetic Retinopathy Study demonstrated that, compared to no treatment, laser photocoagulation treatment resulted in a 50% improvement in visual retention in patients with high-risk proliferative DR (PDR) [4]. Since then, panretinal photocoagulation (PRP) has become the standard treatment for PDR. However, PRP can increase the risk of ME, vitreous hemorrhage, retinal atrophy, burn enlargement, choroidal NV (CNV), and visual field decrease [5,6,7]. Previous studies have shown that 25–43% of eyes treated with PRP develop ME and visual disturbances [5,6,7]. While the exact mechanism underlying ME development after PRP has not been determined, studies have suggested that the release of inflammatory factors after laser irradiation leads to the accumulation of leukocytes, upregulation of angiogenic growth factors such as vascular endothelial growth factor (VEGF), and edematous changes in the retina [8,9].

VEGF induces the formation of abnormal capillaries and consequently plays an important role in the pathogenesis of PDR [10]. Anti-VEGF agents can cause PDR regression and hasten the resolution of diabetic vitreous hemorrhage [11,12]. Several clinical trials have also reported good outcomes of anti-VEGF treatment for ME and retinal NV in patients with diabetes [13,14].

Among the widely used anti-VEGF agents, bevacizumab is a well-known recombinant humanized monoclonal antibody that blocks angiogenesis by inhibiting VEGF-A. It has been successfully used for alleviating ME and NV in patients with PDR [15]. Both PRP and intravitreal bevacizumab (IVB) injection can be useful for PDR treatment, and the effects of bevacizumab and PRP combination therapy have been evaluated in several studies [6,16,17]. However, these studies involved intravitreal injections after PRP. To date, only a few studies have focused on the efficacy of intravitreal anti-VEGF injections before PRP, with conflicting results [17,18,19].

We hypothesized that IVB injection before PRP can prevent the development of ME in patients with PDR. Accordingly, the aim of the present study was to compare the effects of PRP with preoperative IVB injection with those of PRP alone on the prevention of ME in patients with PDR.

## 2. Experimental Section

### 2.1. Patient Enrollment

This study was designed as a retrospective, observational, single-center study. All procedures involving human participants were in accordance with the ethical standards of the institutional and/or national research committee, and with the 1964 Helsinki declaration and its later amendments, or comparable ethical standards. Data for patients with PDR who received PRP between January 2014 and March 2016 were retrospectively reviewed. A systematic evaluation of medical records was performed, and data pertaining to medical history, blood pressure, serum glycosylated hemoglobin (HbA1c) levels, and the findings of complete ocular examinations were reviewed. Ocular examinations included visual acuity measurements (logarithm of the minimum angle of resolution (logMAR)), intraocular pressure measurements, fundus examinations, and CMT measurements performed using spectral domain (SD) optical coherence tomography (OCT) with the Heidelberg OCT device (Heidelberg Engineering, Heidelberg, Germany). Multiple linear scans were processed for obtaining a retinal thickness map. The CMT value reported in the CMT map represented the mean retinal thickness of the macula.

The inclusion criteria were PDR patients with evidence of neovascularization documented on fluorescein angiography. PDR was defined as neovascularization arising from the optic disc and/or retina, which may cause preretinal and vitreous hemorrhage. The exclusion criteria were as follows: lack of preoperative or postoperative OCT findings; history of intraocular surgeries between PRP and IVB injection; severe vitreous hemorrhage; macular hole; epiretinal membrane; active external eye infection; history of thromboembolic events, including myocardial infarction and stroke; systolic and diastolic blood pressures of > 180 and > 110 mmHg, respectively; HbA1c levels of > 15%; visual acuity under 20/200; and history of major surgery within the last month. Patients with clinically significant lens opacity that may affect vision were also excluded from the study.

### 2.2. Study Design

In total, data for 498 patients were retrospectively reviewed. The selected eyes were divided into a combination group, which included those who received IVB injection (1.25 mg/0.05 mL; Avastin^®^, Genentech Inc., San Francisco, CA, USA) before PRP, and a PRP alone group, which included those treated with PRP without IVB injection. The eyes in the combination group received IVB injection one to four weeks before PRP. The criteria for group allocation were decided by the retinal specialist that treated the patients. Visual acuity, intraocular pressure measurements, and fundus examinations were performed before and at one, two, and three months after PRP. Injection-related complications, such as an increase in the intraocular pressure and progression of cataract, were also reviewed.

Approval for this study was obtained from the Gangnam Severance Hospital Institutional Review Board, which provided a waiver of informed consent for the retrospective review of existing patient records (IRB number: 3-2016-0227). The methods adhered to the tenets of the Declaration of Helsinki and were HIPAA compliant.

### 2.3. Intravitreal Injection Procedure

An eyelid speculum was used to stabilize the eyelids. Each eye was prepared using 5% povidone iodine. Bevacizumab (Genentech Inc., South San Francisco, CA, USA) was injected 3.5 mm posterior to the corneal limbus through the pars plana.

### 2.4. OCT Measurements

All eyes underwent OCT before and after treatment at Yonsei University Medical Center, Seoul, Korea. Images of the macula were obtained using an SD-OCT device (Spectralis OCT, Heidelberg Engineering, Franklin, MA, USA). Independent observers performed all measurements. Central thickness maps derived from pre- and post-treatment OCT images were used for CMT calculation.

ME was defined as an accumulation of fluid in the retinal layers around the fovea, resulting in an increased thickening of the retina. Central macular thickness of >300 μm shown in the OCT examination was considered to indicate ME [20].

### 2.5. PRP Procedure

PRP was performed under topical anesthesia with 0.5% proxymetacaine hydrochloride eye drops. A 532 nm green light laser (PASCAL^®^, Topcon Medical Laser Systems, Inc. Santa Clara, CA, USA) with a Mainster Wide Field lens was used for PRP. The inferior and superior areas of the retina were treated in the first and second sessions, respectively. Usually, two sessions were performed in each eye, and a two-week period was maintained between the sessions. OCT was performed after two sessions were completed. Topical anesthetics were used at the time of performing PRP. The spot size was 200 μm, the exposure time was approximately 0.2 s, and the power was adjusted such that a gray–white lesion was produced. Approximately 600–700 laser spots were placed in each session.

### 2.6. Statistical Analysis

All data were statistically analyzed using SPSS 21.0 software (SPSS, Chicago, IL, USA). The values are expressed as means ± standard deviations. Differences between groups were examined by independent t-tests or Wilcoxon signed rank tests. A *p*-value of < 0.05 was considered statistically significant.

## 3. Results

**Figure 1:**
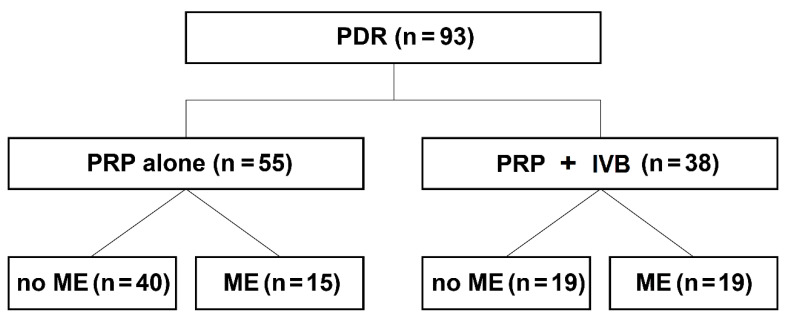
Patient enrollment and study design. In total, 93 eyes of 74 patients with PDR were enrolled. Of these, 55 eyes underwent only PRP and 38 underwent PRP with preoperative IVB injection. Each group was further classified according to the presence of ME at baseline. IVB: intravitreal bevacizumab, ME: macular edema, PRP: panretinal laser photocoagulation, PDR: proliferative diabetic retinopathy.

Of the 498 patients who underwent PRP treatment for PDR, 74 (93 eyes) were considered eligible for our retrospective review. Of the 93 eyes, 55 were included in the PRP alone group and 38 in the combination group (Figure 1). The two groups showed no significant differences in demographic and baseline ocular characteristics except the mean CMT (Table 1). The mean age of participants was 58.7 and 56.6 years in the PRP alone and combination groups, respectively. The mean logMAR visual acuity was 0.246 and 0.311, respectively, while the mean CMT was 277.8 (range = 223 to 353 μm) and 322.9 μm (range = 234 to 652 μm), respectively. In the PRP alone group, there were 15 eyes with ME at baseline. In the combination group, 19 eyes exhibited baseline ME. When the eyes without baseline ME were considered, the mean CMT was 260.3 μm (range = 223 to 296 μm) in the PRP alone group (*n* = 40) and 256.6 μm (range = 234 to 288 μm) in the combination group (*n* = 19), with no significant difference in the logMAR visual acuity (0.262 and 0.226, respectively; *p* = 0.627; Table 1).

### 3.1. Overall Changes in CMT in the Two Groups (PRP Alone: n = 55, Combination: n = 38)

**Figure 2:**
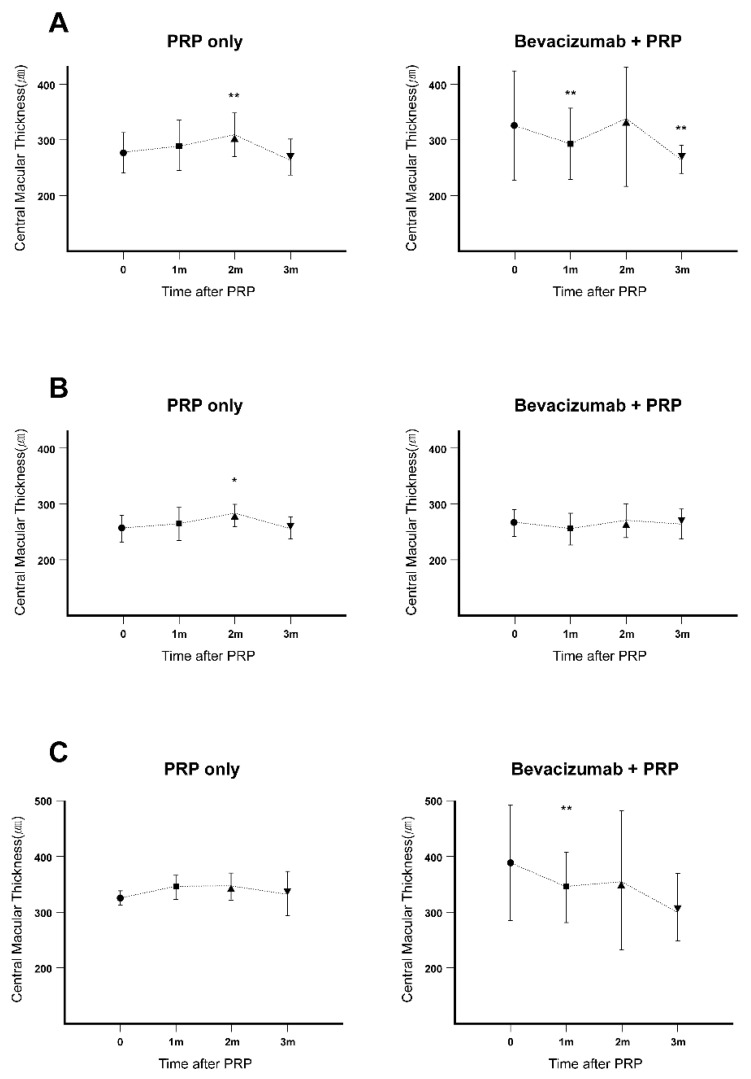
Changes in CMT in patients with PDR who received PRP with or without preoperative IVB injection. The mean CMT was measured using central thickness mapping with OCT at baseline and one, two, and three months after PRP (* *p* < 0.05, ** *p* < 0.01). (**A**) Overall changes in CMT in the two groups. (**B**) Changes in CMT in patients without baseline ME in both groups. (**C**) Changes in CMT in patients with baseline ME in both groups. IVB: intravitreal bevacizumab, PRP: panretinal laser photocoagulation, PDR: proliferative diabetic retinopathy, CMT: central macular thickness, OCT: optical coherence tomography, ME: macular edema.

In the PRP alone group, the mean CMT exhibited a significant increase at two months after PRP. On the other hand, the mean CMT in the combination group exhibited a significant decrease at both one and three months after PRP. In the PRP alone group, the mean CMT increased from 277.8 μm at baseline to 290.4 μm at one month (*p* = 0.201) and 308.8 μm at two months (*p* = 0.002), followed by a decrease to 271.2 μm at three months (*p* = 0.437). In the combination group, the mean CMT was 322.9 μm at baseline, 295.4 μm at one month (*p* = 0.002), 330.1 μm at two months (*p* = 0.906), and 274.5 μm at three months (*p* = 0.030; Figure 2A).

### 3.2. Changes in CMT in the Eyes Without Baseline ME in the Two Groups (PRP Alone: n = 40, Combination: n = 19)

With regard to the eyes without ME at baseline, there was no significant difference in the mean baseline CMT between the PRP alone and combination groups (260.3 μm and 256.6 μm, respectively; *p* = 0.509). In the PRP alone group, the mean CMT significantly increased at two months after PRP. In contrast, the mean CMT in the combination group showed no significant changes during the follow-up period. In the PRP alone group, the mean CMT at baseline and one, two, and three months after PRP was 260.3, 267.0 (*p* = 0.080), 281.8 (*p* = 0.024), and 261.9 (*p* = 0.778) μm, respectively; these values were 266.1, 255.4 (*p* = 0.162), 269.0 (*p* = 0.960), and 263.8 (*p* = 0.055) μm, respectively, in the combination group (Figure 2B).

### 3.3. Changes in CMT in Eyes with Baseline ME in the Two Groups (PRP Alone: n = 15, Combination: n = 19)

The mean CMT in eyes with baseline ME exhibited a significant increase at one month after PRP in the PRP alone group and a significant decrease at one month after PRP in the combination group. In the PRP alone group, the mean CMT was 323.3 μm at baseline and 350.0 (*p* = 0.031), 345.9 (*p* = 0.107), and 327.0 (*p* = 0.750) μm at one, two, and three months after PRP, respectively. In the combination group, these values were 386.2, 341.7 (*p* = 0.009), 353.9 (*p* = 0.956), and 339.0 (*p* = 0.926; Figure 2C) μm, respectively.

### 3.4. Changes in Visual Acuity

**Figure 3:**
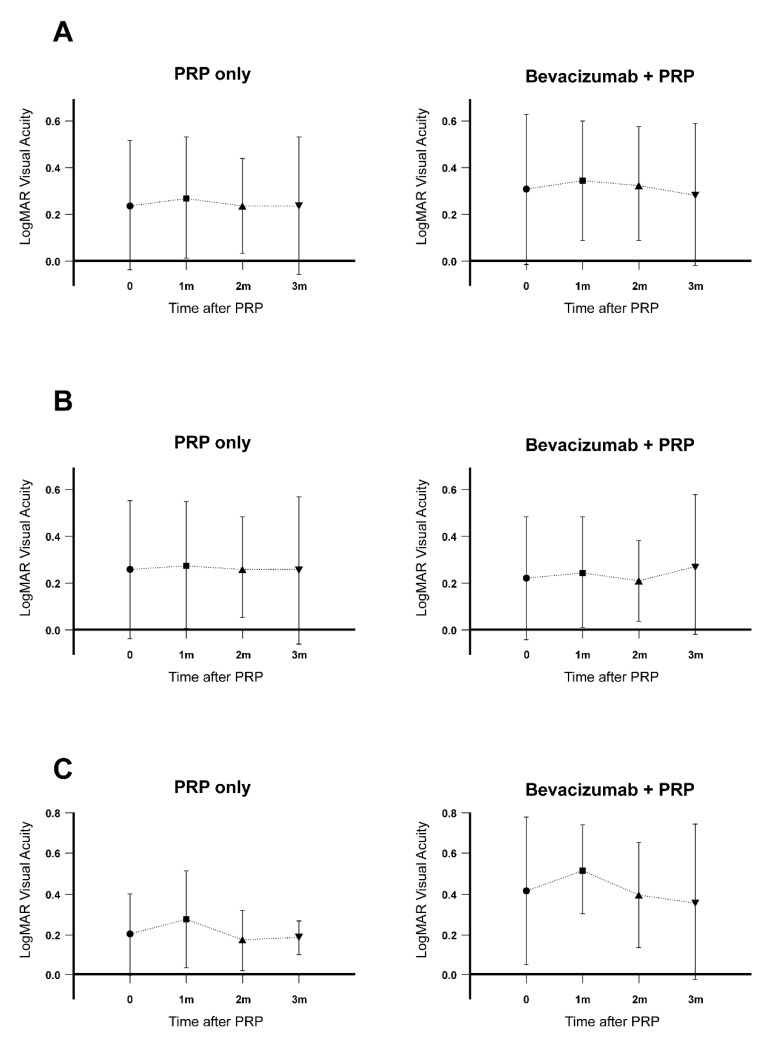
Visual acuity changes in patients with PDR who received PRP with or without preoperative IVB injection. Visual acuity was measured at baseline and one, two, and three months after PRP. (**A**) Overall changes in the logMAR visual acuity in the two groups. (**B**) Changes in the logMAR visual acuity in patients without baseline ME in both groups. (**C**) Changes in the logMAR visual acuity in patients with baseline ME in both groups. IVB: intravitreal bevacizumab, PRP: panretinal laser photocoagulation, PDR: proliferative diabetic retinopathy, logMAR: logarithm of the minimum angle of resolution, ME: macular edema.

Visual acuity changes at all time points were comparable between the PRP alone and combination groups, regardless of the presence of baseline ME. Although there were no statistically significant differences, all groups showed the same trend of a decrease during the first month after treatment and recovery thereafter (Figure 3).

### 3.5. Injection-Related Complications

No significant adverse events were recorded during the follow-up period. None of the eyes showed significant cataract progression or endophthalmitis during the follow-up period. Moreover, there were no statistically significant differences in the follow-up intraocular pressure measurements between the two groups (data not shown). No systemic adverse events were recorded for any patient.

## 4. Discussion

In the present study, we found that IVB injection before PRP prevented ME development and even reduced existing ME in patients with PDR.

Currently, PRP is one of the most important treatment modalities for PDR. The Early Treatment Diabetic Retinopathy Study reported that early photocoagulation lowers the risk of severe vision loss after five years by 23% [3]. While PRP can salvage the patient’s vision, it is associated with side effects, such as the development of ME [5,6,7]. In the present study, CMT increased when PRP was performed alone and decreased when PRP was preceded by IVB injection. ME induced by PRP was also observed, consistent with findings in previous reports [6,7,21]. Interestingly, IVB injection before PRP was also found to reduce existing ME up to three months after PRP. Moreover, bevacizumab injection is relatively cheaper than aflibercept or ranibizumab injections [22,23]. Thus, adjunctive bevacizumab injection before PRP can serve as a good, cost-effective treatment.

We also found that our results were influenced by the presence of ME before PRP. For eyes without baseline ME, CMT during the follow-up period remained similar in the combination group and increased in the PRP alone group. On the other hand, when ME was present at baseline, combination treatment led to a significant decrease in CMT. Thus, IVB injection can be used before PRP even if ME is present at baseline, considering it showed the ability to not only prevent the development of ME, but also treat existing ME.

The effects of IVB injection before PRP observed in the present study can be attributed to several reasons. Even though PRP is an established therapy for the regression of NV, several studies have observed negative effects on retinal structure and function [24,25]. During PRP, the retinal pigment epithelium is the principal site for laser energy absorption, and the subsequent damage spreads to the retina. After PRP treatment, cytokines, such as VEGF, interleukin-6 (IL-6), and RANTES (CCL5), are upregulated. These cytokines may be the primary contributors to ME immediately after PRP [26,27,28,29]. VEGF plays an important role in the breakdown of the blood–retinal barrier. This breakdown leads to an increase in vascular permeability and fluid leakage, eventually resulting in the development of ME [30]. VEGF also induces vessel dilation and increases the ocular blood flow. Therefore, inhibition of VEGF by bevacizumab may reduce the retinal vascular permeability and decrease fluid leakage. Although VEGF decreases over time after PRP, it does not decrease immediately after the procedure [27,31]. This may have attributed to the favorable effects of preoperative IVB injection in our study. In the group with ME at baseline, IVB may have reduced the existing ME via its anti-VEGF effects in the early phase, which overwhelm the effects of other cytokines. Alternatively, IVB may have offset the newly developing ME in the group without ME at baseline. Unlike CMT, however, we did not find a significant difference in visual acuity changes after treatment between the two groups. This may be due to the small sample size or the short follow-up period.

This study had some limitations, including the retrospective design, relatively small sample size, short-term follow-up period, and the fact that the baseline CMT was different between the combination and PRP only groups. However, when the eyes were classified according to the presence or absence of ME at baseline, those with similar CMT and the absence of ME at baseline still demonstrated favorable outcomes after PRP with preoperative IVB injection. Additionally, the fact that the exact time for IVB injection was not the same (IVB injection was performed one to four weeks before PRP) and that the study included patients with poor glycemic control may have affected the study results. Hence, large, randomized prospective studies are necessary to overcome these limitations and confirm our results. Moreover, further studies should determine the optimal timing for IVB injection before PRP, because no studies have assessed this parameter to date.

In summary, we found that IVB injection before PRP leads to decreased CMT in comparison to CMT in patients with PRP alone. These findings suggest that IVB injection prior to PRP may be an effective adjunctive modality for the prevention of ME after PRP or the treatment of existing ME in patients with PDR.

## Figures and Tables

**Table 1:** Baseline clinical characteristics of patients with proliferative diabetic retinopathy who underwent panretinal photocoagulation with (combination group) or without (PRP only group) preoperative intravitreal bevacizumab injection (1.25 mg/0.05 mL).

Characteristics	PRP Only Group			Combination Group			*p*-Value
Presence of ME at baseline	No ME	ME	Combined	No ME	ME	Combined	No ME/ME/Combined
Number of patients	30	11	41	15	18	33	
Number of eyes	40	15	55	19	19	38	
Sex (Male/Female)	13/15	10/3	23/18	7/8	14/4	21/12	
Age, mean (SD) years	58.9 (6.78)	57.3 (8.48)	58.7 (7.27)	56.8 (16.71)	54.9 (13.29)	56.6 (14.35)	0.550/0.569/0.436
HbA1c	8.18	7.33	7.78	8.44	6.83	8.14	0.739/0.430/0.704
BP systolic (mmHg)	140	135	138	128	133	131	0.095/0.452/0.261
BP diastolic (mmHg)	79	82	80	81	74	78	0.501/0.109/0.146
Mean baseline CMT	260.3 µm	323.3 µm	277.8 µm	256.6 µm	382.2 µm	322.9 µm	0.509/0.149/0.001
Mean baseline V/A (logMAR)	0.262	0.197	0.246	0.226	0.408	0.311	0.627/0.335/0.319

ME: macular edema, PRP: panretinal laser photocoagulation, PDR: proliferative diabetic retinopathy, BP: blood pressure, CMT: central macular thickness, HbA1c: glycosylated hemoglobin, V/A: visual acuity, SD: standard deviation; comparisons were performed using t-tests.
